# Type 2 Diabetes Mellitus Impairs the Reverse Transendothelial Migration Capacity (rTEM) of Inflammatory CD14^+^CD16^−^ Monocytes: Novel Mechanism for Enhanced Subendothelial Monocyte Accumulation in Diabetes

**DOI:** 10.3390/cells14191567

**Published:** 2025-10-09

**Authors:** Dilvin Semo, Adama Sidibé, Kallipatti Sanjith Shanmuganathan, Nicolle Müller, Ulrich A. Müller, Beat A. Imhof, Rinesh Godfrey, Johannes Waltenberger

**Affiliations:** 1 Vascular Signalling, Molecular Cardiology, Department of Cardiology I—Coronary and Peripheral Vascular Disease, Heart Failure, University Hospital Münster, 48149 Münster, Germany; 2 Department of Pathology and Immunology, Centre Médical Universitaire (CMU), Medical Faculty, University of Geneva, Rue Michel-Servet 1, CH-1211 Geneva, Switzerland; 3 Department of Internal Medicine III, University Hospital Jena, 07743 Jena, Germany; 4 Practice for Endocrinology and Diabetes, MVZ Dr. Kielstein GmbH Erfurt, 07743 Jena, Germany; 5 Department of Physiology, Cardiovascular Research Institute Maastricht (CARIM), 6229 ER Maastricht, The Netherlands; 6 Department of Cardiovascular Medicine, Medical Faculty, University of Münster, 48149 Münster, Germany; 7 Hirslanden Klinik im Park, Cardiovascular Medicine, Diagnostic and Therapeutic Heart Center AG, 8002 Zürich, Switzerland

**Keywords:** diabetes mellitus, monocytes, vascular dysfunction, adhesion, reverse transendothelial migration (rTEM), Junctional Adhesion Molecule 3 (JAM-3), Macrophage-1 antigen (MAC-1)

## Abstract

Background: Type 2 diabetes mellitus (DM) is a major cardiovascular risk factor that induces monocyte dysfunction and contributes to their accumulation in atherosclerotic lesions. Monocyte recruitment and accumulation in the tissues contribute to chronic inflammation and are essential to the pathobiology of diabetes-induced atherosclerosis. However, the mechanisms that drive the accumulation of monocytes in the diabetic environment are not clearly understood. Methods: Primary monocytes from type 2 (T2) DM and non-T2DM individuals were isolated using magnet-assisted cell sorting. To examine the influence of a diabetic milieu on monocyte function, monocytes from T2DM patients, db/db mice, or human monocytes subjected to hyperglycaemia were analysed for their responses to pro-atherogenic cytokines using Boyden chamber assays. Furthermore, the interactions of non-diabetic and diabetic monocytes with TNFα-inflamed endothelium were studied using live-cell imaging under physiological flow conditions. RT-qPCR and FACS were used to study the expression of relevant molecules involved in monocyte-endothelium interaction. Results: CD14^+^CD16^−^ monocytes isolated from T2DM patients or monocytes exposed to hyperglycaemic conditions showed reduced chemotactic responses towards atherosclerosis-promoting cytokines, CCL2 and CX3CL1, indicating monocyte dysfunction. Under flow conditions, the transendothelial migration (TEM) capacity of T2DM monocytes was significantly reduced. Even though these monocytes adhered to the endothelial monolayer, only a few transmigrated. Interestingly, the T2DM monocytes and monocytes exposed to hyperglycaemic conditions accumulated in the ablumen following transendothelial migration. The time period in the ablumen of T2DM cells was prolonged, as there was a significant impairment of the reverse transendothelial migration (rTEM). Mechanistically, the T2DM milieu specifically induced the activation of monocyte integrins, Macrophage-1 antigen (Mac-1; integrin αMβ2 consisting of CD11b and CD18), and Lymphocyte function-associated antigen 1 (LFA-1; αLβ2 consisting of CD11a and CD18). Furthermore, elevated levels of CD18 transcripts were detected in T2DM monocytes. Junctional Adhesion Molecule 3 (JAM-3)–MAC-1 interactions are known to impede rTEM and T2DM milieu-potentiated JAM-3 expression in human coronary artery endothelial cells (HCAEC). Finally, the overexpression of JAM-3 on HCAEC was sufficient to completely recapitulate the impaired rTEM phenotype. Conclusions: Our results revealed for the first time that the enhanced T2DM monocyte accumulation in the ablumen is not secondary to the elevated transmigration through the endothelium. Instead, the accumulation of monocytes is due to the direct consequence of a dysfunctional rTEM, potentially due to enhanced JAM3-MAC1 engagement. Our results highlight the importance of restoring the rTEM capacity of monocytes to reduce monocyte accumulation-dependent inflammation induction and atherogenesis in the T2DM environment.

## 1. Introduction

The International Diabetes Federation estimates that among the 415 million people with diabetes, 91% of them have type 2 diabetes mellitus (T2DM). It is predicted that type 2 diabetes mellitus (T2DM) is a prevalent, complex metabolic disease that leads to hyperglycaemia [1]. Cardiovascular disease (CVD) is a major cause of morbidity and mortality among people with T2DM [2]. Diabetes mellitus is responsible for a 2–4-fold rise in the development of coronary artery disease (CAD) [3,4]. The main contributing factor to the development of vascular disease is the build-up of plaques in the arteries, termed atherosclerosis. Leukocytes, in general, and monocytes, in particular, play a vital role in the induction of the inflammatory response that promotes atherogenesis [5,6]. Circulating monocytes adhere to the inflamed endothelium, transmigrate, and infiltrate the vessel wall, engulf oxLDL to become foam cells, and participate positively in the development and progression of atherosclerosis [7].

An increased number of classical monocytes (CD14^+^CD16^−^) in the blood is directly correlated with cardiovascular events independent of sex, age, and classical cardiovascular risk factors [8]. Accumulation of CD14^+^CD16^−^ monocytes in the infarct border zone has been reported in patients who eventually succumb to myocardial infarction [9]. Blood monocytosis is a predominant feature in most atherosclerosis mouse models [10]. The recruitment of monocytes from the blood into the atherosclerotic lesions is a very orchestrated process involving several receptors involved in adhesion and transmigration. Numerous studies have focused on the role of CCR2-CCL2, CX3CR1-CX3CL1, and CCR5-CCL5 chemokine receptor interactions in the monocyte recruitment-dependent atheroprogression [11].

Furthermore, monocyte recruitment to the plaques is dependent on the extent of inflammation-dependent activation of the endothelium, primarily through the increased expression of cell adhesion molecules such as endothelial vascular cell adhesion molecule-1 (VACM-1) and intercellular adhesion molecule 1 (ICAM-1) [12,13]. The outside-in signalling induced in the leukocytes by the interaction of integrins like αLβ2, consisting of CD11a and CD18 or integrin or lymphocyte function-associated antigen-1 (LFA-1), αMβ2 integrin consisting of CD11a and CD18 or macrophage integrin-1 (Mac-1) and α4β1 integrin or very late antigen-4 (VLA-4) to ICAM-1 results in both leucocyte and endothelial activation [14,15]. For the transmigration to happen, the interactions of LFA-1 and VLA-4 with ICAM-1 and VCAM-1 promote cell adhesion molecule clustering and VE-Cadherin dissociation-dependent loosening of tight junctions [16]. Alterations in the expressions of the molecules mentioned above could contribute to enhanced monocyte recruitment during atheroprogression [17,18]. It is well established that monocytes are mainly involved in atherosclerosis development, and targeting monocyte activation, transmigration, and accumulation is considered a therapeutic approach to reducing tissue inflammation and plaque development [19]. However, it is still unclear whether the enhanced migration of monocytes is primarily responsible for the higher monocyte/macrophage accumulation in the plaque.

Recent studies have shown that monocytes can re-enter the circulation by re-navigating back through the endothelium in the reverse direction [20]. This is a less characterised—although crucial—mechanism that regulates monocyte retention in the abluminal compartment following transmigration. This process, termed reverse transendothelial migration (rTEM), is also considered essential for macrophage emigration from the plaque. Very early studies have shown that as the plaque progresses, the emigration of macrophages from the plaque is reduced [21]. Therefore, enhanced retention of monocytes/macrophages could contribute to their higher numbers and promote inflammation-dependent atherogenesis and atheroprogression. While T2DM conditions are known to dampen the monocyte recruitment process through the induction of monocyte dysfunction, making monocytes refractory to growth factor stimulation [22,23], the T2DM environment potentiates monocyte-dependent atherosclerosis development. Therefore, it is entirely likely that T2DM conditions interfere with the rTEM of monocytes and accelerate their accumulation. Such a possibility has never been investigated, and we have focused on this aspect in this study.

## 2. Results

### 2.1. T2DM Conditions Do Not Specifically Modulate the Chemotactic Responses of CD14^+^CD16^−^ Monocytes Towards Atherosclerosis-Promoting Cytokines

**Figure 1:**
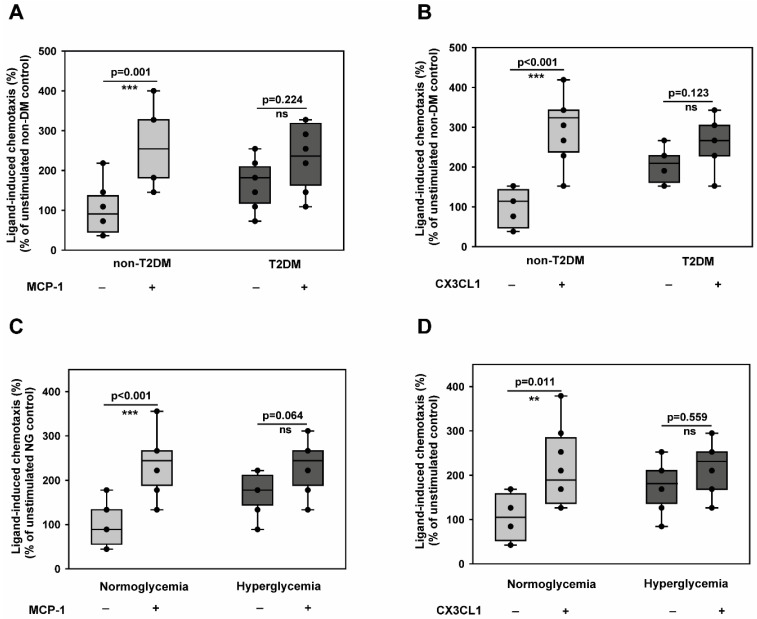
Type 2 diabetes mellitus impairs directional chemotactic responses of CD14^+^CD16^−^ monocytes toward atherosclerosis-promoting cytokines while enhancing random motility. (**A**,**B**) CD14^+^CD16^−^ inflammatory monocytes isolated from non-diabetic control individuals (*n* = 6) or T2DM patients (*n* = 6) were analysed for their migratory responses using Boyden chamber assays. Chemokinesis (random motility in the absence of stimulus) and chemotaxis (directional migration toward stimulus) were quantified in response to pro-atherogenic cytokines MCP-1 (**A**) and CX3CL1 (**B**). Data show both absolute cell counts (left *y*-axis) and chemotactic index (stimulated/unstimulated migration ratio, right *y*-axis). T2DM monocytes exhibited significantly enhanced chemokinesis but reduced chemotactic responsiveness to both stimuli. (**C**,**D**) CD14^+^CD16^−^ inflammatory monocytes from healthy donors were subjected to hyperglycaemic conditions (25 mM glucose) or normoglycemic control conditions (5 mM glucose + 20 mM mannitol osmotic control) for 48 h, then analysed for migratory responses as in (**A**,**B**). Hyperglycaemic treatment recapitulated the T2DM phenotype with enhanced baseline motility but impaired directional chemotaxis toward MCP-1 (**C**) and CX3CL1 (**D**). *n* = 6 independent experiments. All data are means ± SEM. Statistical comparisons: ** *p* < 0.01, *** *p* < 0.001 by the Mann–Whitney U test.

Since it is known that monocyte accumulation promotes atherogenesis and creates a pro-inflammatory state, we wondered whether diabetic conditions accelerate monocyte chemotaxis towards athero-promoting cytokines like CCL2 (MCP-1) and CX3CL1 (fractalkine). This was particularly important to understand given the reported induction of monocyte dysfunction in T2DM [23]. To investigate that, we carried out monocyte static transmigration assays using Boyden chamber assays. As shown in Figure 1A,B, monocytes from T2DM patients were indeed in an activated state, as reflected by their enhanced random motility (chemokinesis). However, we could not detect any acceleration of the chemotactic activity towards MCP-1 (Figure 1A) and CX3CL1 (Figure 1B) by T2DM monocytes. Furthermore, we established an in vitro hyperglycaemic setting that mimics diabetic conditions by exposing monocytes to 25 mM of glucose for 48 h. Under these conditions, neither the survival (Supplementary Figure S1A) nor the expression of the monocyte-specific marker, CD14, was altered (Supplementary Figure S1B).

Furthermore, the in vitro hyperglycaemic monocytes were pro-inflammatory, as evidenced by the upregulation of both TNFα and IL-6 transcripts (Supplementary Figure S1C). This indicated that there is no role for apoptosis or spontaneous differentiation during in vitro culture, and the in vitro culture conditions impart a pro-inflammatory phenotype to monocytes, as described before [24]. Using this model, we demonstrated that the T2DM phenotype could be recapitulated by monocytes exposed to in vitro hyperglycaemia for 48 h (Figure 1C,D). These findings indicate that diabetic conditions could induce monocyte activation but do not potentiate their transmigration towards CCL2 and CX3CL1.

### 2.2. CD14^+^CD16^−^ Monocytes Exposed to Hyperglycaemic Conditions Do Not Exhibit an Enhanced Transmigration Phenotype

**Figure 2:**
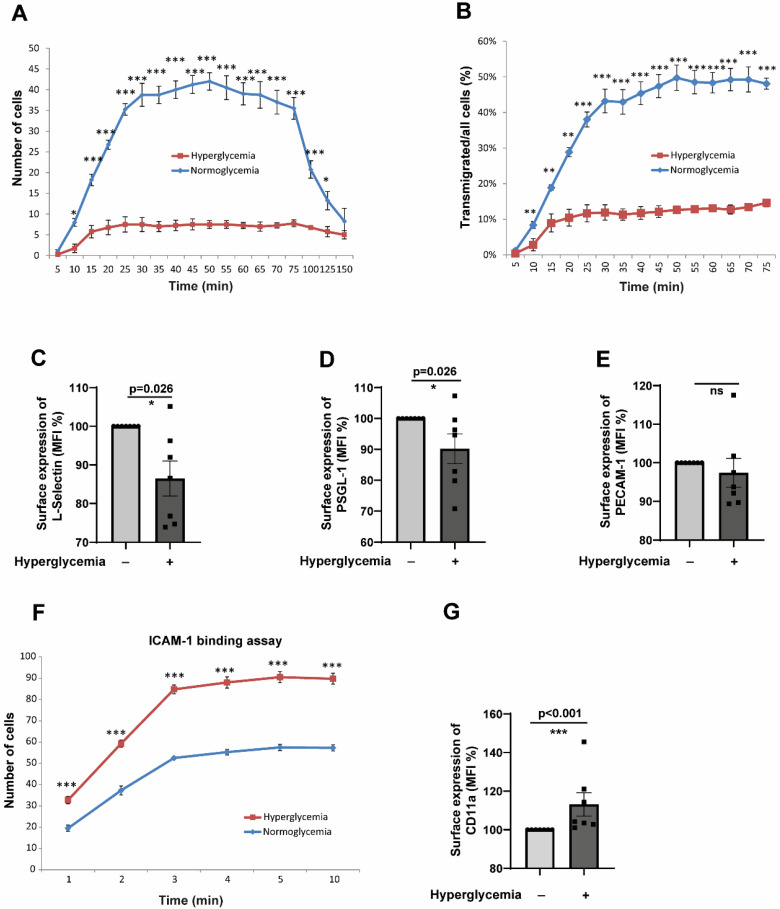
Hyperglycaemic conditions significantly inhibit transendothelial migration of CD14^+^CD16^−^ monocytes despite enhanced ICAM-1 binding capacity. (**A**) Time-course analysis of transendothelial migration under physiological flow conditions (0.12 mL/min). CD14^+^CD16^−^ monocytes pre-exposed to hyperglycaemic (25 mM glucose) or normoglycemic control conditions (5 mM glucose + 20 mM mannitol) for 48 h were flowed over TNFα-activated HUVECs. Individual monocytes were tracked, and their positions marked at regular intervals (5–150 min). Hyperglycaemic monocytes showed significantly reduced transmigration rates compared to normoglycemic controls, with differences evident from 10 to 125 min (representative of 5 independent experiments, * *p* < 0.05 at multiple time points). (**B**) Quantitative analysis of transmigration efficiency normalised to total adherent cells to control for adhesion differences. Hyperglycaemic monocytes demonstrated a 30–40% reduction in transmigration capacity (normoglycaemic: 48.2 ± 6.7% vs. hyperglycaemic: 28.9 ± 4.3% of adherent cells transmigrated, *n* = 5, *p* < 0.01). (**C**–**E**) Surface expression analysis of key adhesion molecules by flow cytometry after 48 h treatment. Hyperglycaemic conditions significantly reduced L-selectin (**C**) and PSGL-1 (**D**) expression (MFI: L-selectin control 145.2 ± 12.3 vs. hyperglycaemic 89.7 ± 8.9, *p* < 0.01; PSGL-1 control 198.5 ± 18.4 vs. hyperglycaemic 134.2 ± 15.1, *p* < 0.05), while PECAM-1 levels remained unchanged (**E**). *n* = 7 per group. (**F**) ICAM-1 binding assay under flow conditions. Paradoxically, hyperglycaemic monocytes showed significantly enhanced adhesion to ICAM-1-coated surfaces compared to normoglycemic controls (representative of 5 experiments, *p* < 0.01), indicating increased binding capacity despite reduced transmigration. (**G**) Flow cytometric analysis revealed elevated CD11a expression in hyperglycaemic monocytes (MFI: control 87.3 ± 7.2 vs. hyperglycaemic 124.8 ± 11.5, *n* = 7, *p* < 0.05), explaining the enhanced ICAM-1 binding capacity. All data are means ± SEM. Statistical comparisons: * *p* < 0.05, ** *p* < 0.01, *** *p* < 0.001 by the Mann–Whitney U test.

The interaction of primary monocytes and endothelial cells was investigated during live cell imaging under flow conditions for several hours. Tracking the monocytes disclosed several impairments in their functional parameters. We carried out flow assays to understand whether the impaired chemotactic behaviour of monocytes exposed to hyperglycaemic conditions (described in Figure 1C,D) results in an impairment of monocyte transmigration. Under flow, we analysed the ability of hyperglycaemic monocytes to undergo transendothelial migration through human umbilical vein endothelial cells (HUVECs). As shown in Figure 2A, hyperglycaemic conditions significantly impaired the transmigration capacity of monocytes. The difference in the transmigration behaviour was visible from 10 to 125 min. Besides that, we also analysed the number of transmigrated cells. We correlated that to the total number of adherent cells to rule out the differences in adhesion to the observed lower transendothelial migration rates. When all the adherent cells were analysed, the hyperglycaemic adherent monocytes showed almost a 30–40% reduction in the transmigration capacity compared to normoglycemic monocytes (Figure 2B). Therefore, it can be concluded that the alterations in the transendothelial migration exhibited by the hyperglycaemic monocytes seen under flow conditions are due to the phenotypic alterations induced by hyperglycaemia. Since leukocyte-endothelial interaction and transendothelial migration are governed by a plethora of ligand-receptor interactions [25], we looked at the expression levels of selectins, PSGL-1 (P-Selectin glycoprotein ligand-1), L(eukocyte)-Selectin, and PECAM-1 (Platelet–endothelial cell adhesion molecule-1). As shown in Figure 2C,D, our experiments revealed a significantly reduced expression level of L-Selectin and PSGL-1 in hyperglycaemic monocytes. PSGL-1, a dimer expressed on leukocytes, interacts with L-, P(latelet)- and E(ndothelial)-selectins. PSGL1 plays a vital role in rolling arrest and signalling processes [26]. It is tempting to speculate that a lower expression of selectins leads to a decreased capacity of the cells to escape the blood flow and initiate the initial engagement with the endothelial cell receptors, promoting transendothelial migration. PECAM-1, a member of the immunoglobulin superfamily, interacts with VCAM-1 (vascular cell adhesion molecule-1), which is only expressed on the endothelial cell surface and allows an arrest of rolling, is required for a firm adhesion and also for transendothelial migration [27]. However, we did not detect significant changes in the PECAM-1 level in hyperglycaemic monocytes (Figure 2E). Intercellular Adhesion Molecule 1 (ICAM-1) is an essential mediator of the leukocyte adhesion process by binding to the β2 integrin lymphocyte function-associated antigen (LFA)-1 (αLβ2 or CD11a-CD18) on the surface of leukocytes [28]. Therefore, we wondered whether ICAM-1 interactions with LFA-1 are impaired in hyperglycaemic monocytes. ICAM-1 binding assays revealed that hyperglycaemic monocytes can significantly adhere more to ICAM-1 compared to normoglycaemic monocytes (Figure 2F). This could be due to the upregulation of integrins. We, therefore, checked the expression levels of CD11a, CD11b, and CD18. As expected, the higher adhesion ability of hyperglycaemic monocytes was due to elevated expression of CD11a (Figure 2G). However, we could not detect any differences in the expression levels of CD18 or CD11b (Supplementary Figure S2A,B).

### 2.3. CD14^+^CD16^−^ Monocytes Exposed to Hyperglycaemic Conditions Exhibit a Very Pronounced Reverse Transmigration Defect

**Figure 3:**
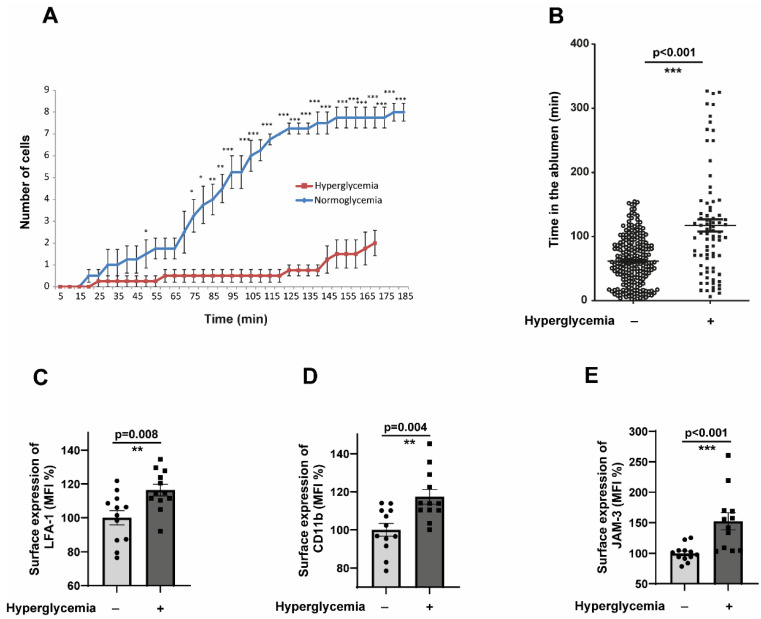
CD14^+^CD16^−^ monocytes exposed to hyperglycaemic conditions exhibit a very pronounced reverse transmigration defect. (**A**) Time-course analysis of reverse transendothelial migration (rTEM) kinetics under physiological flow conditions. CD14^+^CD16^−^ monocytes pre-conditioned under normoglycemic (5 mM glucose + 20 mM mannitol osmotic control) or hyperglycaemic (25 mM glucose) conditions for 48 h were flowed over TNFα-activated HUVECs and allowed to adhere and transmigrate for 5 min. Following this initial transmigration phase, individual monocytes were tracked during the reverse migration process, with positions recorded at 15 min intervals from 5 to 185 min post-initial flow. (**B**) Same as in (**A**), but the analysis was performed in such a way that the time spent by each cell in the ablumen was calculated. Normoglycemia, *n* = 225; hyperglycaemia, *n* = 79 (**C**,**D**). CD14^+^CD16^−^ monocytes were exposed to either normoglycemic or hyperglycaemic conditions for 48 h. The cells were then analysed by flow cytometry for the surface expression of activated LFA-1 and CD11b. *n* = 7. The mean fluorescence intensity (MFI) was then quantified. All data are means ± SEM. (**E**) Human Coronary Artery Endothelial cells (HCAECs) were exposed to normoglycemic (5 mM Glucose + 20 mM Mannitol) or hyperglycaemic conditions (25 mM Glucose) for 48 h. Afterwards, HCAECs were stained with anti-human JAM-C antibodies and analysed by flow cytometry. The mean fluorescence intensity (MFI) was then quantified. *n* = 6. All data are means ± SEM. * *p* < 0.05, ** *p* < 0.01, and *** *p* < 0.001.

The results from Section 2.2 collectively indicate that hyperglycaemic conditions do not impart a higher transmigration capacity to monocytes, and there should be other ways by which monocytes accumulate in the ablumen. Therefore, we hypothesised that alterations in the reverse transendothelial migration (rTEM) process could result in the accumulation of monocytes without enhancing transendothelial migration. We investigated whether hyperglycaemic monocytes exhibit such an aberrant phenotype. Flow assays revealed that hyperglycaemic monocytes are significantly impaired in their rTEM. Transmigrated normoglycemic monocytes started to reverse transmigrate after 45 min, whereas hyperglycaemic monocytes were delayed significantly in undergoing reverse transmigration and took almost 155 min to reverse transmigrate (Figure 3A).

Furthermore, we specifically looked at the time duration at which monocytes remain in the ablumen. Hyperglycaemic monocytes spend significantly more time in the ablumen than their normoglycemic counterparts (Figure 3B). Reverse transmigration of monocytes is known to be controlled by the interactions of Junctional Adhesion Molecule 3 (JAM-3), by acting as a counterreceptor for the integrin MAC-1 (αMβ2; CD11b-CD18). JAM-3 has been validated as a cognate counterreceptor for MAC-1 [29]. Supplementary Figure S2A,B showed that both CD18 and CD11b expression were not altered in hyperglycaemic monocytes. As an alternate approach, we analysed the activation status of CD11b and LFA-1 integrins. Interestingly, both LFA-1 and CD11b were heavily activated in hyperglycaemic monocytes (Figure 3C,D). We also analysed the expression of JAM-3 in the endothelial cells subjected to hyperglycaemic conditions. The results revealed a statistically significant upregulation of JAM-3 (Figure 3E). These results suggested that the upregulated JAM-3 on the surface of endothelium could interact with activated CD11b on monocytes, leading to the entrapment of monocytes in the ablumen under hyperglycaemic conditions.

### 2.4. T2DM CD14^+^CD16^−^ Monocytes Recapitulate the Impaired TEM and rTEM Phenotype

**Figure 4:**
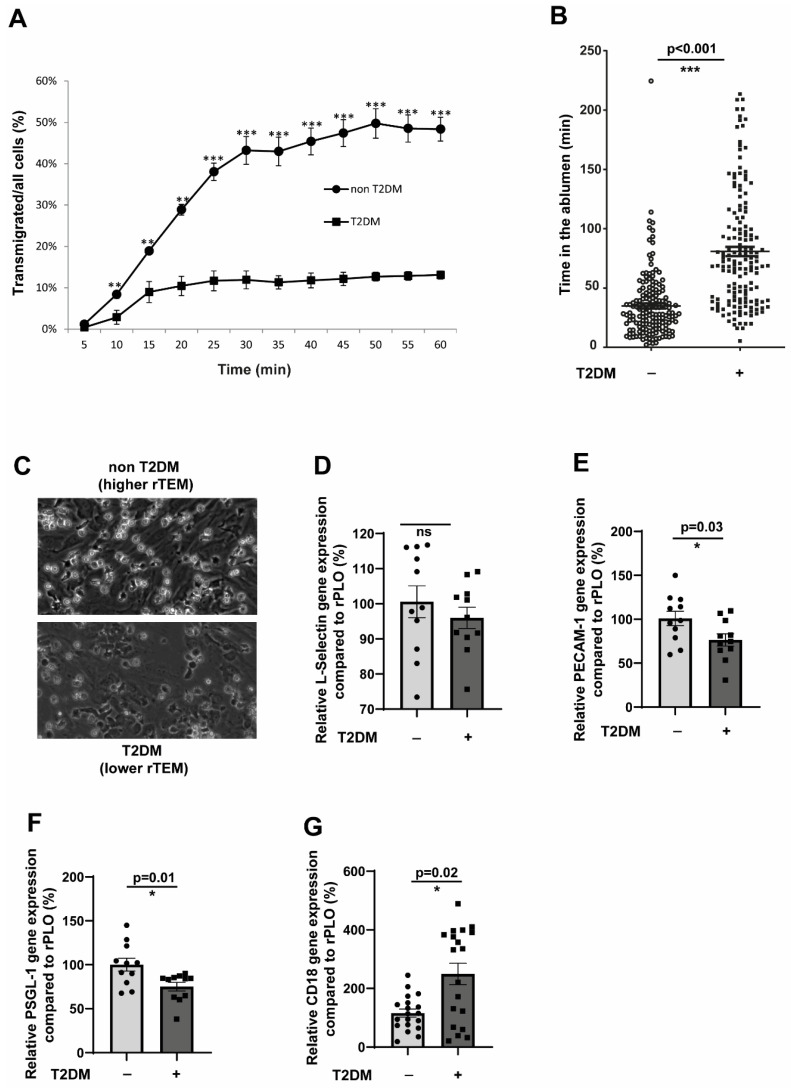
Inflammatory CD14^+^CD16^−^ monocytes from T2DM patients recapitulate the impaired TEM and rTEM phenotype. (**A**) CD14^+^CD16^−^ monocytes from non-T2DM individuals (*n* = 3) or T2DM patients (*n* = 6) were flowed over activated HUVECs for 5 min. Monocytes were individually tracked, and their positions were marked at regular intervals (5–60 min). (**B**) Same as in (**A**), but the analysis was performed in such a way that the time spent by each cell in the ablumen was calculated. Non-T2DM, *n* = 166; T2DM, *n* = 160. (**C**) Representative images from the experiment (**A**). (**D**–**F**) CD14^+^CD16^−^ monocytes from non-T2DM individuals (*n* = 11) or T2DM patients (*n* = 11) were analysed for the expression of L-selectin, PECAM-1, and PSGL-1 using RT-qPCR. All data are means ± SEM. (**G**) CD14^+^CD16^−^ monocytes from non-T2DM individuals (*n* = 22) or T2DM patients (*n* = 21) were analysed for the expression of L-selectin, PECAM-1, and PSGL-1 using RT-qPCR. All data are means ± SEM. * *p* < 0.05, ** *p* < 0.01, and *** *p* < 0.001.

Since all the abnormalities displayed by the monocytes in terms of EC cell interactions, as described in the previous results, were tested in in vitro hyperglycaemic conditions, we used monocytes from T2DM patients. T2DM monocytes readily exhibited an impaired transmigration phenotype, similar to the behaviour of hyperglycaemic monocytes (Figure 4A). Only 10% of the adherent T2DM monocytes transmigrated compared to around 50% of the non-T2DM monocytes. Furthermore, T2DM monocytes accumulated robustly in the ablumen and remained in the ablumen for a significantly extended period (Figure 4B). The reverse transmigrating non-T2DM monocytes are readily detectable (Figure 4C). We then analysed the molecules responsible for such behaviour. Unlike hyperglycaemic monocytes, we did not detect aberrant alterations in the L-selectin levels (Figure 4D). However, PSGL-1 and PECAM-1 showed significantly lower expression levels in T2DM monocytes (Figure 4E,F). This partly explains the impaired TEM phenotype of T2DM monocytes. Interestingly, T2DM monocytes displayed an elevated expression of two chemokine receptors important for transmigration, CCR2 and CX3CR1 (Supplemental Figure S3A,B). Unlike selectins, the critical member of the LFA-1 and MAC-1 integrins, the CD18 expression, became significantly upregulated in T2DM monocytes.

### 2.5. JAM-3 Is the Primary Regulator of Impaired rTEM Phenotype

**Figure 5:**
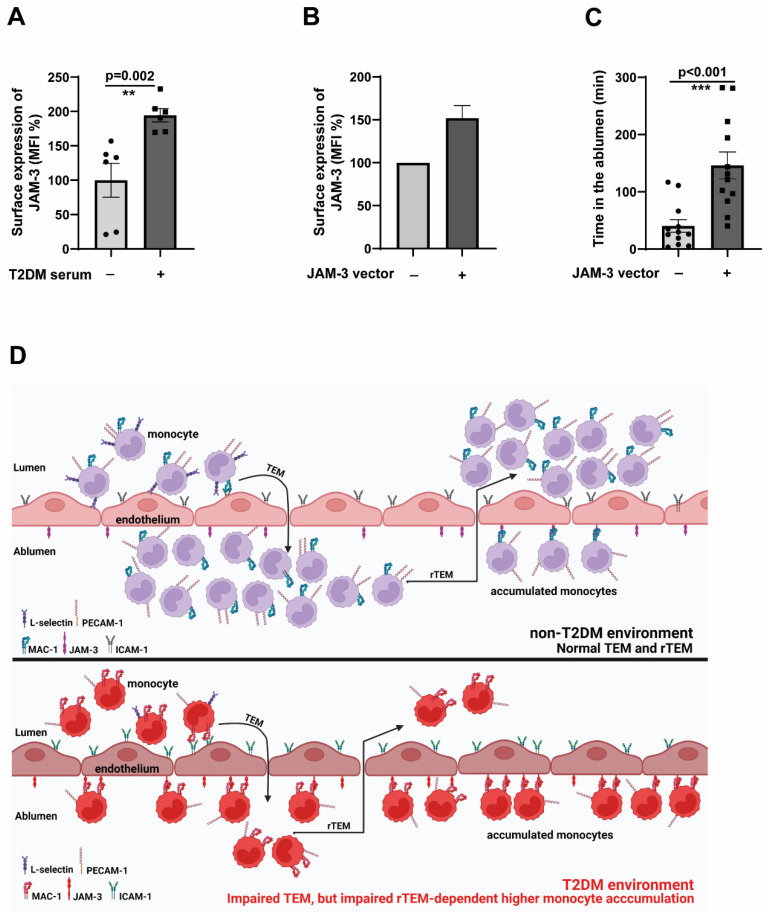
JAM-3 is the primary regulator of impaired rTEM phenotype (**A**). HCAECs (Human Coronary Artery Endothelial Cells) were seeded until 80% confluence and then exposed to a 1:5 serum: cell culture medium mix for 48 h. The serum was obtained from either non-T2DM individuals or T2DM patients after incubation with HCAECs, and analysed by flow cytometry for the surface expression of JAM-3. The mean fluorescence intensity (MFI) was then quantified. *n* = 6. All data are means ± SEM. (**B**) HCAECs were electroporated with either JAM-3 overexpression plasmid or control plasmid, and after 24 h, the expression levels of JAM-3 were analysed using flow cytometry. (**C**) HCAECs were electroporated with either JAM-3 overexpression plasmid or control plasmid. After 24 h, CD14^+^CD16^−^ monocytes obtained from healthy donors were flowed over JAM-3 overexpressing or control HCAECs for 5 min. Monocytes were then individually tracked, and their positions were marked at regular intervals (5–250 min). The time spent by each cell in the ablumen was then calculated. All data are means ± SEM. ** *p* < 0.01, and *** *p* < 0.001. (**D**) The working model depicts the mechanisms that mediate impaired TEM and rTEM of monocytes, resulting in a higher accumulation of monocytes in the ablumen. Reduced L-selectin and PECAM-1 potentially mediate impaired TEM, and the upregulation of activated MAC-1-dependent engagement of JAM-3 in endothelial cells traps monocytes in the ablumen, preventing these cells from reverse transmigrating. Accumulated monocytes will result in higher inflammation induction and promote the atherogenesis process.

Figure 3E demonstrated significant upregulation of JAM-3 expressions in endothelial cells in hyperglycaemic conditions. We wondered whether the T2DM milieu would also contribute to JAM-3 expression. To that end, we used serum from T2DM patients and stimulated endothelial cells for 48 h. Under these conditions, we detected a significantly elevated JAM-3 expression (Figure 5A). Since the major binding partner of JAM-3, the MAC-1 complex, is strongly associated with adhesion, we could not manipulate the levels of MAC-1 to understand its specific role in mediating rTEM, which will significantly impair the adhesion and transmigration process.

To investigate how relevant the expression induction of JAM-3 is in mediating an impaired rTEM, we overexpressed JAM-3 in endothelial cells. The overexpression was adjusted to be around 50% more than the control cells (Figure 5B). Confirming the specific role of JAM-3 in mediating rTEM, JAM-3 overexpressed endothelial cells displayed significantly higher retention in the ablumen (Figure 5C) compared to the controls. These data collectively validate the pivotal role of JAM-3 in regulating the impaired rTEM of monocytes.

## 3. Discussion

Accumulation of monocytes in the subendothelial space and subsequent differentiation into macrophages and foam cell formation is a significant determinant for atherosclerotic lesion formation [30]. The higher the accumulation of inflammatory monocytes in the ablumen, the more the inflammation is enhanced severalfold. Recruitment of monocytes and retention within atherosclerotic lesions is widely considered to contribute to plaque development [31]. It is known that the blockade of monocytes and the aberrant recruitment of monocytes could reduce the atherosclerotic burden [32]. Even though T2DM monocytes display impaired transmigration and dysfunction [22], T2DM conditions result in monocyte accumulation and predispose the individual to CAD [33]. It has been known for quite some time that the progression of atherosclerotic plaques may result from robust monocyte recruitment into arterial walls and reduced emigration of monocytes from existing lesions [21]. Therefore, monocyte retention is also critical for atheroprogression. Even though almost all the steps in the monocyte recruitment process have been unravelled, the exact mechanisms that govern the egress are unknown. Understanding the factors that control the egress of monocytes/macrophages from the plaque may constitute a viable strategy for the regression of advanced plaques.

Our findings reveal several seemingly paradoxical behaviours in T2DM monocytes that actually support a unified mechanism of altered monocyte function. First, T2DM monocytes exhibit enhanced chemokinesis (random motility) while simultaneously showing reduced chemotaxis (directed migration toward specific cytokines). This apparent contradiction reflects the well-established concept that excessive random motility interferes with cells’ ability to sense and respond appropriately to directional chemotactic gradients, as previously demonstrated in various inflammatory conditions [22,34,35,36]. Second, while hyperglycaemic monocytes show enhanced binding to ICAM-1 in static assays, they demonstrate reduced transendothelial migration under dynamic flow conditions. This discrepancy highlights the critical difference between static adhesion capacity and functional migration under physiological flow, where multiple coordinated steps, including rolling, firm adhesion, and transmigration, must occur sequentially. The enhanced integrin activation we observed may actually represent dysfunctional ‘sticky’ monocytes that bind more avidly but migrate less efficiently, contributing to their retention rather than facilitating their transit.

Reverse transendothelial migration (rTEM) of monocytes is a rather underinvestigated area. Though several seminal studies were carried out on the rTEM of neutrophils [37,38], data from monocytes remain obscure. rTEM is particularly relevant for the retention of monocytes. If rTEM is defective, there is a higher chance of monocytes being retained in the ablumen, promoting atherosclerosis development. The only study that investigated this validated the role of JAM-3 in retaining monocytes and pointed out that JAM-3 blockade could be an approach to promote monocyte/macrophage egress [39]. The extent to which T2DM conditions influence the rTEM process is entirely unknown. To our knowledge, this is the first study investigating the impact of T2DM on the rTEM of monocytes.

The finding that hyperglycaemic and T2DM monocytes are not responding robustly to athero-promoting cytokines CX3CL1 and MCP-1 seems counterintuitive. However, it is very clear from the studies in our laboratory that monocytes from T2DM, CAD, and in vitro hypercholesterolemic conditions show significant impairment in their chemotactic responses to MCP-1, PlGF-1, or VEGF-A [22,34,35,36]. This is due to the alterations in the intracellular signalling cascades due to higher oxidative stress, leading to the preactivation of several kinases [22,23]. The finding that the transendothelial migration of hyperglycaemic and T2DM monocytes is impaired is also provocative. This data could indicate that there will be reduced monocyte recruitment, and then how could T2DM conditions promote atherosclerosis with less efficient monocyte recruitment? However, several studies have shown that atherosclerosis development is only partially based on how many monocytes are recruited. How many transmigrated monocytes are retained in the ablumen to potentiate inflammation is also important. From that perspective, our data demonstrate that T2DM and hyperglycaemic monocytes accumulate robustly in the ablumen compared to non-T2DM and normoglycemic monocytes.

The expression pattern of L-selectin, PSGL-1, and PECAM-1 seems aberrant in both hyperglycaemic and T2DM monocytes. Although L-selectin is an adhesion molecule primarily known for its role in the tethering/rolling of leukocytes, recent studies have implicated L-selectin in the TEM process by regulating monocyte protrusion [40,41]. Similarly, PECAM-1 is a potent regulator of TEM. Blockade of PECAM-1 using blocking antibodies has reduced TEM of monocytes by 70–90% [27,42,43]. Our results are consistent with this published notion that any impairment in L-selection and PECAM-1 could directly influence the TEM of monocytes. Understanding how T2DM conditions contribute to suppressing these two integral molecules requires further studies.

Our experimental strategy employed a dual approach combining isolated hyperglycaemia with validation using complete diabetic serum to strengthen clinical relevance. The hyperglycaemic model (25 mM glucose) allowed us to isolate glucose-specific effects while controlling for osmolarity, establishing hyperglycaemia as sufficient to induce the observed phenotypes. The subsequent validation with T2DM patient serum confirmed that these effects occur under clinically relevant conditions where multiple diabetic factors (dyslipidaemia, inflammatory cytokines, advanced glycation end products, and altered protein profiles) act in concert. This approach was intentionally designed as translational validation rather than a mechanistic dissection of serum components, as T2DM patients are exposed to the complete diabetic milieu rather than isolated hyperglycaemia. While further characterisation of the active serum factors would provide additional mechanistic insight, our findings demonstrate that the JAM-3 upregulation and subsequent rTEM impairment occur under both isolated hyperglycaemic conditions and the complex diabetic environment, supporting the clinical relevance of our in vitro observations.

The retention of T2DM and hyperglycaemic monocytes within the ablumen due to impaired transmigration is a fascinating and novel finding of this study. Compared to non-T2DM or normoglycemic monocytes, T2DM monocytes get stuck within the ablumen by engaging with the JAM-3 through MAC-1, unable to reverse transmigrate, allowing a higher accumulation of monocytes even if the transmigration process is slightly attenuated. Monocyte retention is aided by the expression upregulation of JAM-3 on the endothelial surface, upregulation of CD18, and activation of CD11b on the monocyte surface (Figure 5D). This could contribute to higher inflammation and atherogenesis in the T2DM environment. Higher emigration of monocytes from atherosclerotic lesions was shown to promote regression of established plaques [21].

Similarly, impaired emigration could lead to progressive plaque formation. Taking this information into perspective, it seems highly likely that in T2DM conditions, impaired rTEM could play a vital role in the development of atherosclerosis. We have also identified the importance of JAM-3 in retaining monocytes in the ablumen, which is consistent with the published data [20,39]. Elevating the levels of JAM-3 alone in endothelial cells could recapitulate the impaired rTEM phenotype even when monocytes from a healthy donor were used. This indicated that JAM-3 is integral in mediating monocytic rTEM. Interestingly, JAM-C also represents a pathologically relevant target in oncology. Anti-JAM-3 antibodies are currently being tested to reduce tumour burden in haematological malignancies [44] and could be evaluated in an atherosclerosis setting. Several other molecules could control rTEM of leukocytes. Osteopontin has been reported to regulate monocyte recirculation and aid in their retention [45]. Further investigations are required to clarify the role of other relevant molecules that could regulate the rTEM of monocytes in the T2DM environment.

## 4. Materials and Methods

### 4.1. Monocyte Isolation from Clinical Cohorts and Healthy Individuals

CD14^+^CD16^−^ human monocytes were isolated from healthy donors and non-T2DM individuals or T2DM patients according to a published protocol [23] using magnet-assisted cell sorting (MACS) and negative selection using Monocyte Isolation Kit II human from Miltenyi Biotec (North Rhine-Westphalia, Germany). The study was approved by the scientific and ethics committee of the University of Münster and conforms to the principles of the Declaration of Helsinki. The blood bank obtained written informed consent from all donors, and thrombocyte reduction filters were provided anonymously without sharing personal and detailed information. FACS confirmed the purity of isolated cells, which were around 98% pure. The patient characteristics are described in detail in Supplementary Table S1.

### 4.2. Monocyte Culture

Primary human monocytes were maintained in RPMI-1640 medium (+L-Glutamine,—D-Glucose, Thermo Scientific, Waltham, MA, USA) supplemented with 5 mM Glucose, 10% foetal bovine serum (FBS), and 1% Penicillin/Streptomycin. For migration experiments and signalling studies, cells were starved for 2–4 h in FBS-free medium. Monocytes were kept in an incubator at 37 °C and 5% CO_2_. Normoglycemic medium: RPMI-1640 medium (Gibco^®^, Thermo Fisher Scientific, Waltham, MA, USA), Penicillin/Streptomycin, 5 mM glucose, and 20% foetal bovine serum. Hyperglycaemic medium: RPMI-1640 medium (Gibco^®^, Thermo Scientific, Waltham, MA, USA) Penicillin/Streptomycin, 30 mM glucose, and 20% foetal bovine serum.

### 4.3. Monocyte Chemotaxis

Chemotaxis assays were performed as described previously [22,36], using a 48-well Boyden chamber (Neuroprobe, Gaithersburg, MD, USA) and Nucleopore PET membrane (Whatman^®^, Maidstone, UK) with 5 µm diameter pores. Cells in a concentration of 0.5 × 10^6^ cells/mL were allowed to migrate for 90 min at 37 °C and 5% CO_2_. The cells that migrated through the pores were counted. For quantification, migrated cells were counted by 20 high-power fields in four wells using the Axioskop 2 Plus microscope (Carl Zeiss, Jena, Germany).

### 4.4. Preparation of HUVEC Monolayer for Flow Assays

HUVECs (human umbilical vein endothelial cells) from the same batch and at the same passage number (P4 for assays with pre-treated primary monocytic cells or P1 for frozen patient primary monocytic cells) were cultured and frozen at −80 °C until usage. Ibidi µ-Slide VI, 0.4 slides (Ibidi, Gräfelfing, Germany) for flow experiments, were coated with PBS (phosphate-buffered saline), 0.1 mg/mL collagen G (Type I-collagen, Sigma-Aldrich, Taufkirchen, Germany), 0.2% gelatin. A total of 25,000 to 30,000 HUVECs per chamber were seeded on the slides and incubated at 37 °C, 10% CO_2_ until a confluent monolayer was built. HUVECs were cultured in M199—20% FBS—1% penicillin/streptomycin. Monolayers were activated for seven hours with 1000 Units TNFα, diluted in M199—20% FBS—1% Penicillin/Streptomycin before experiments.

### 4.5. Fow Assay Experiments with Primary Monocytes

An experimental flow setup was prepared by connecting Ibidi µ-Slide VI 0.4, according to the manufacturer’s references, to a reservoir of M199 (Gibco^®^)—Penicillin/Streptomycin and to a pump (NE—1000, New Era, Farmingdale, NY, USA). The slides were kept in an incubating system with 37.0—37.4 °C and 5% CO_2_ during the experiment. Flow rates were set at 0.12 mL/min (flow rates in post-capillary venules) and controlled by the pump. TNFα was washed away from the monolayer before adding 1.5 × 10^6^ primary monocytic cells (in a concentration of 5 × 10^6^ cells/mL)—either pre-cultured in normoglycemic or hyperglycaemic conditions.

The experimental setup was performed similarly for flow assay experiments with frozen patient primary monocytic cells with a few modifications. The HUVEC monolayer was prepared with HUVECs at passage number 1 from the same donor. Frozen patients’ primary monocytic cells and controls were cultured for 2 h in a normoglycemic medium (5 mM glucose) and at 37 °C/5% CO_2_ before being added to the flow system. M199 (Gibco^®^)—penicillin/streptomycin—20% FBS was used for flow assays. During flow experiments, one picture was taken every 15 s with the AxioVert 200-microscope (Carl Zeiss, Jena, Germany) with 10× magnification for eight to nine hours. Data generated during flow experiments were analysed with Fiji Image J. Four equally sized parts of the pictures were analysed using the plugin for a manual count. Only adherent cells were counted and tracked during adhesion steps, transendothelial migration, and reverse migration.

### 4.6. Preparation of HUVEC Monolayer for Flow Assays Flow Assay Experiments with Frozen Patient Primary Monocytic Cells

The experimental setup was performed following the flow assay experiments with pre-treated primary monocytic cells. The HUVEC monolayer was prepared with HUVECs at passage number 1 from the same donor. Frozen patients’ primary monocytic cells and controls were cultured for 2 h in a normoglycemic medium (5 mM glucose) and at 37 °C/5% CO2 before being added to the flow system. M199 (Gibco, Waltham, MA, USA)—Penicillin/Streptomycin—20% FBS was used for flow assays.

### 4.7. Human Coronary Artery Endothelial Cell (HCAEC) Culture and Genetic Manipulation Experiments

Cells were obtained from Lonza (Basel, Switzerland) and were maintained in EBMTM-2 Basal Medium (CC-3156) and EGMTM-2 MV Microvascular Endothelial Cell Growth Medium SingleQuots^TM^ supplements (CC-4147) as per the recommendation of the manufacturer. Overexpression of JAM-3 in HCAECs was carried out using electroporation using the Lonza nucleofector device as per the manufacturer’s instructions.

### 4.8. FACS Staining and Analysis

FACS was performed according to the manufacturer’s standard protocols. In short, 1.5–2.0 × 10^6^ Monocytes from the experiments were taken and washed once with 1× ice-cold PBS and fixed with 80% Methanol. Staining was carried out in FACS buffer (PBS with 1% BSA and 0.1% sodium azide) for 30 min at 4 °C using antibodies mentioned below. After staining, the cells were washed with 1× PBS, and FACS analysis was performed using Guava easyCyte (Millipore, Burlington, MA, USA). The following antibodies were from Miltenyi Biotech, Bergisch Gladbach, Germany were used: CD14 (CD14-FITC, human (clone: TÜK4), 130-098-058), L-Selectin (CD62L-PE, human (clone:145/15)), PSGL—1 (CD162-FITC, human (clone: REA319)), PECAM-1 (CD31-FITC, human (clone: REA730)), CX3CR1 8 Anti-CX3CR1-PE, human (clone: 2A9-1), 130-099-618, CCR2 (CD192 (CCR2)-PE, human (clone: REA264), 130-103-902, CD323 JAM-3 (Anti-JAM3-PE, human (clone REA429), 130-106-510), CXCR4 (Anti-CX3CR1-PE, human (clone: 2A9-1), 130-099-618), CD18 (CD18-FITC, human (clone: TS1/18), 130-101-242), CD11a (CD195 (CCR5)—PE, Cat.-No. 560932, BD Biosciences, Heidelberg, Germany), CD11b (Alexa Fluor^®^ 488 anti-human CD11b Antibody, Cat. No. 301318, BioLegend, San Diego, CA, USA), activated CD11b (FITC anti-human CD11b (activated), (clone: CBRM1/5) BioLegend, San Diego, CA, USA)), activated LFA-1 (Alexa Flour^®^ 488 anti-human CD11a/CD18 (LFA-1), (clone: m24) BioLegend, San Diego, CA, USA).

### 4.9. Cell Viability Assays

The CellTiter 96R AQueous Non-Radioactive Cell Proliferation Assay (Promega, Darmstadt, Germany) was used to measure monocyte viability/proliferation/metabolic activity according to the manufacturer’s protocol. Briefly, cells (1 × 10^4^ cells/well) were seeded into 96-well plates in normoglycemic or hyperglycaemic conditions for 48 h. At the different time points, MTS/PMS (20:5) was added for 1 h. Metabolically active cells convert MTS (4.5-dimethylthiazol-2-yl)-5-(3-carboxymethoxyphenyl)-2-(4-sulfophenyl)-2H-tetrazolium) in the presence of PMS (phenazine methosulfate) to Formazan. Formazan was measured using the Victor X3 (Perkin Elmer, Waltham, MA, USA) at an absorbance of 490 nm.

### 4.10. ICAM-1 Binding Assay

Ibidi µ-Slide VI 0.4 slides (Ibidi) were coated with PBS/5 µg/mL ICAM-1 at four °C overnight and activated for two hours with PBS/0.5% BSA-solution for two hours at room temperature. Three normoglycemic or hyperglycaemic pre-treated cells were added to the Ibidi slides, 1.5 × 10^6^ primary monocytic cells (in a concentration of 5 × 10^6^ cells/mL). After 90 min of incubation, the slides were connected to a flow system (flow rate 0.12 mL/min). The adhesion of the cells to ICAM-1 was observed by taking a picture all 15 s with the AxioObserver 200—microscope (Carl Zeiss, Jena, Germany) for 15 min. Data were analysed using the manual count plugin of the Fiji Image J software.

### 4.11. RNA Isolation and qPCR

For the extraction of RNA, roughly 5–8 × 10^6^ monocytes were used routinely. For in vitro experiments, the RNA was extracted between 8 and 12 h post-cell treatment. Total RNA purification was performed using the NucleoSpin RNA isolation kit (Macherey-Nagel, Dueren, Germany), and cDNA was synthesised using the RevertAid First Strand cDNA Synthesis Kit (Thermo Scientific, Waltham, MA, USA). qPCR was carried out using iTaq™ Universal SYBR^®^ Green supermix (Bio-Rad, Hercules, CA, USA) in the Connect Real-Time PCR Detection System (Bio-Rad, Hercules, CA, USA). Each sample’s threshold cycle (Cq) value was calculated, and the expression of target gene mRNA relative to rplO was determined by the 2^−ΔΔCt^ method. The sequences of the primers used can be found in Supplementary Table S2.

### 4.12. Statistical Analysis

To analyse the significance of differences in experiments with monocytes isolated from diabetic or healthy individuals/mice, the Mann–Whitney Rank Sum Test (for intergroup comparisons) or Kruskal–Wallis One-Way Analysis of Variance on Ranks with Tukey or Dunn’s post hoc correction was used. For all the other experiments, two-sample independent t-tests or when multiple comparisons were made, Kruskal–Wallis One-Way Analysis of Variance on Ranks with Tukey or Dunn’s post hoc correction was performed. SigmaPlot software was used for the statistical analysis. The level of significance was defined as *p* < 0.05. Graphs for flow assay and ICAM-1 binding assay underflow were created using Microsoft Excel software. The average of four equally sized parts of the pictures was used for graphs and statistics. All other statistics and graphs were generated using GraphPad Prism 8 software.

## 5. Conclusions

In conclusion, using the cell culture model of in vitro hyperglycaemia and T2DM monocytes, we uncovered that T2DM conditions impair transendothelial migration (TEM) and reverse transendothelial migration (rTEM). This led to the enhanced retention of monocytes in the ablumen even when transmigration rates were compromised. Mechanistically, TEM impairment was due to the defect in L-selectin and PECAM-1 expression, whereas the rTEM defect was due to the enhanced interactions between JAM-3 and MAC-1. These data demonstrate a previously unrecognised mechanism through which T2DM promotes inflammatory monocyte accumulation, an important step for atherogenesis. Furthermore, our results highlight the importance of restoring the rTEM capacity of monocytes to reduce monocyte accumulation-dependent inflammation induction and atherogenesis in the T2DM environment.

## Data Availability

The original contributions presented in the study are included in the article/Supplementary Material, further inquiries can be directed to the corresponding authors.

## Supplementary Materials

The following supporting information can be downloaded at https://www.mdpi.com/article/10.3390/cells14191567/s1, Figure S1: In vitro hyperglycemic conditions do not induce toxicity or impair its inflammatory properties. Figure S2: Expression pattern of integrins by hyperglycemic monocytes. Figure S3: Expression pattern of chemokine receptors in T2DM monocytes. Table: S1: Clinical characteristics of the non-T2DM and T2DM individuals used in the study. Table: S2: RT-qPCR primer sequences used in the study.
