# Supplementary material for: Type 2 Diabetes Mellitus Impairs the Reverse Transendothelial Migration Capacity (rTEM) of Inflammatory CD14+CD16− Monocytes: Novel Mechanism for Enhanced Subendothelial Monocyte Accumulation in Diabetes
Source: Cells. 2025 Oct 9;14(19):1567. doi: 10.3390/cells14191567 (PMC12523990; doi:10.3390/cells14191567)
Supplement: Supplementary file 1 [file cells-14-01567-s001.zip › Supplementary Tables.pdf]

Supplementary Table S1

Clinical characteristics of the non-T2DM and T2DM individuals used in the study

	Non-DM	T2 DM	significance
N	12	12	
Age (years)	56±17.8	63±7.2	n.s.
Sex (male/female)	9/3	8/4	n.s.
BMI (kg/m^2)	26.5±3.2	36.9±4.9	<0.001
HbA1c (%)	5.33±0.25	8.64±1.02	<0.001
Glucose (mmol/L)	5.06±0.44	12.7±4.75	<0.001
Smoking (yes/no)	1/11	1/11	n.s.
Hypercholesterolaemia (yes/no)	6/6	0/12	0.006

Supplementary Table S2

RT-qPCR primer sequences used in the study

**Gene**	**Forward primer sequence (5' -3')**	**Reverse primer sequence (5' -3')**
**hCCR-2**	ATGCTGTCCACATCTCGTTCTCG	TTATAAACCAGCCGAGACTTC CTGC
**hCD18**	ACTGCGAGTGTGACACCATCA	GGACTTGAGCTTCTCCTTCTC
**hCD11a**	AAATGGAAGGACCCTGATGCTC	TGTAGCGGATGATGTCTTTGG C
**hCD11b**	CAGATCAACAATGTGACCGTATGG G	CATCATGTCCTTGTACTGCCGC TTG
**hCX3CR1**	TGACTGGCAGATCCAGAGGTT	GTTTTCTGTCACTGATTCAGGG AA
**hL-selectin**	TCACAGTGTGCCTTCAGCTGCT	TCTGGTGCTGATAGAGGCTCA C
**hPSGL-1**	TGACACCACTCCTCTGACTGGG	CTCCATAGCTGCTGAATCCGT G
**hPECAM-1**	GCAGAGTACCAGGTGTTGGT	GAACAGTTGACCCTCACGAT
**hrplO**	AATCTCCAGGGGCACCATT	CGCTGGCTCCCACTTTGT
