# Supplementary material for: Type 2 Diabetes Mellitus Impairs the Reverse Transendothelial Migration Capacity (rTEM) of Inflammatory CD14+CD16− Monocytes: Novel Mechanism for Enhanced Subendothelial Monocyte Accumulation in Diabetes
Source: Cells. 2025 Oct 9;14(19):1567. doi: 10.3390/cells14191567 (PMC12523990; doi:10.3390/cells14191567)
Supplement: Supplementary file 1 [file cells-14-01567-s001.zip › Supplementary Figures.pdf]

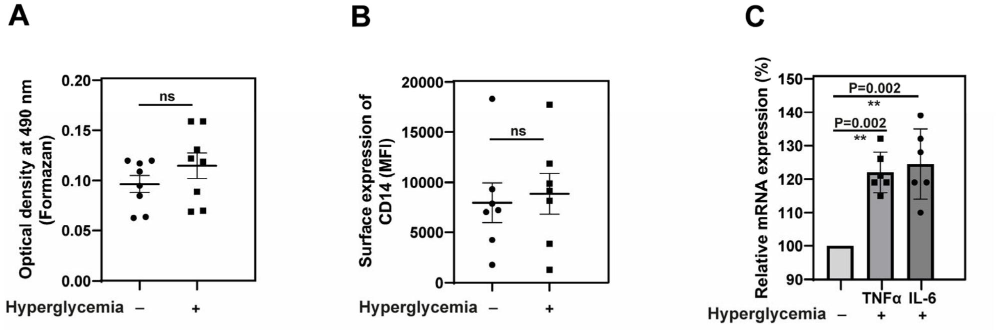

Figure S1. In vitro hyperglycemic conditions do not induce toxicity or impair its inflammatory properties (A) CD14 ++CD16 - monocytes exposed to either normoglycemic or hyperglycemic conditions for 48 hours. After that, the proliferation or metabolic activity of these cells were analyzed using a modified MTT assay. The resultant generation of Formazan by metabolically active cells was measured using a spectrophotometer at an absorbance of 490 nm. n=8. All data are means ± SEM (n=8). (B) CD14 ++CD16 - monocytes exposed to either normoglycemic or hyperglycemic conditions for 48 hours. The cells were then analysed by flow cytometry for the surface expression of monocyte marker, CD14. The mean fluorescence intensity (MFI) was then quantified. All data are means ± SEM. n=7. (C) CD14 ++CD16 - monocytes exposed to either normoglycemic or hyperglycemic conditions for 48 hours. Thereafter, the cells were analysed for the expression of TNFα and IL-6 using RT-qPCR. All data are means ± SEM. n=6 . **p < 0.01. Supplementary

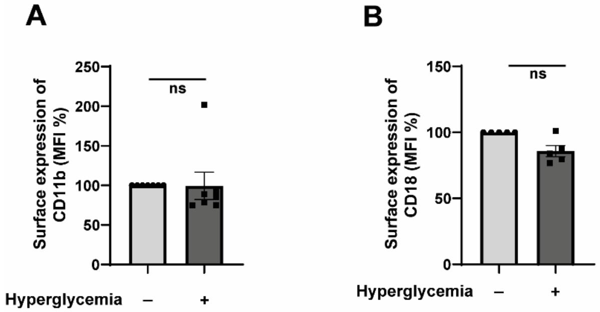

Figure S2. Expression pattern of integrins by hyperglycemic monocytes (A) CD14 ++CD16 - monocytes exposed to either normoglycemic or hyperglycemic conditions for 48 hours. The cells were then analysed by flow cytometry for the surface expression of integrin CD11b. The mean fluorescence intensity (MFI) was then quantified. All data are means ± SEM. n=5. (B) CD14 ++CD16 - monocytes exposed to either normoglycemic or hyperglycemic conditions for 48 hours. The cells were then analysed by flow cytometry for the surface expression of integrin CD18. The mean fluorescence intensity (MFI) was then quantified. All data are means ± SEM. n=5. n.s= non-significant. Supplementary

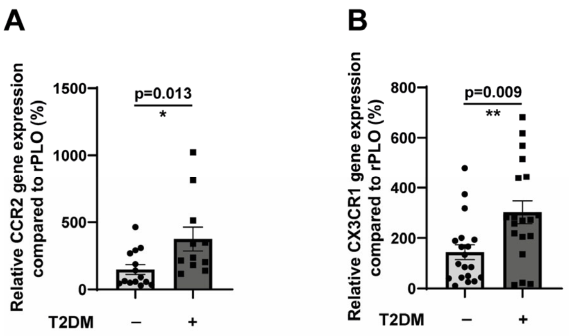

Figure S3. Expression pattern of chemokine receptors in T2DM monocytes (A) CD14 ++CD16 - monocytes from non-T2DM individuals (n=11) or T2DM patients (n=11) were analysed for the expression of CCR2 using RT-qPCR. All data are means ± SEM. (B) CD14 ++CD16 - monocytes from non-T2DM individuals (n=16) or T2DM patients (n=17) were analysed for the expression of chemokine receptor CX3CR1 using RT-qPCR. All data are means ± SEM. *p < 0,05, **p < 0.01.
